# BMI, Dental Caries, and Risk Factors among Elementary School Children: A Cross-Sectional Study

**DOI:** 10.3390/children11091145

**Published:** 2024-09-21

**Authors:** Deema J. Farsi

**Affiliations:** Department of Pediatric Dentistry, Faculty of Dentistry, Kind Abdulaziz University, P.O. Box 80200, Jeddah 21589, Saudi Arabia; dfarsi@kau.edu.sa; Tel.: +966-12-640-2000 (ext. 20388)

**Keywords:** dental caries, BMI, obesity, child, physical activity, diet, KIDMED

## Abstract

**Background/Objectives:** Obesity and dental caries are multifactorial diseases with high prevalence amongst Saudi children. The aim of the study was to determine their association with common risk factors. **Methods:** This cross-sectional study examined 300 children attending elementary schools. After taking their height and weight, their body mass indices (BMIs) were calculated. Oral examination was performed to record the decayed, missed, and filled scores (dmft/DMFT). A thorough questionnaire was compiled and validated to gather information on diet and physical activity (PA). The KIDMED score was calculated from a 16-item questionnaire to assess diet. Junk food and PA scores were also calculated based on relevant questions. Nonparametric tests were used to assess the associations between the scores and health outcomes (dmft/DMFT and BMI). **Results:** Fifty-eight of the children had poor diets, which was associated with higher dmft/DMFT (*p* = 0.012). PA levels were very low, and the average PA score was 2.12 ± 0.61. No association was found between BMI percentiles and PA level, nor between BMI percentiles and diet quality. Older children had lower BMI percentiles compared with younger children (coefficient = −9.35, 95% CI: −17.05, −1.65), and a borderline significant negative association was observed between dmft/DMFT and BMI percentile. **Conclusions:** Poor diets and chips consumption were related to dental caries. Obesity was not related to diet quality nor PA level.

## 1. Introduction

There are several means of defining obesity. The World Health Organization (WHO) defines it as “abnormal or excessive fat accumulation that may impair health” [1]. Another explanatory definition is “an excess amount of body fat in relation to lean body mass, caused when energy intake far outweighs energy expenditure” [2]. A person with a body mass index (BMI) at or greater than 30 is considered obese [1]. The long-term health consequences of obesity are physical as well as psychological. It has been linked to type 2 diabetes, sleep apnea, cardiovascular disease, fatty liver disease, hypertension, orthopedic disease, and low self-esteem [3,4,5,6].

Childhood obesity is a serious neurobehavioral health issue, affecting as many as 41 million children worldwide [7]. The etiological factors for childhood obesity are diverse and complex [8]. The obesogenic environment, which increases an individual’s risk of obesity, is linked to factors such as the absence of safe, convenient opportunities for daily physical activity (PA) and limited access to affordable healthy food [9]. Obesity has also been linked to behavioral habits that are predetermined genetically or acquired during the course of life. For example, emotional stress can lead to obesity if the child practices maladaptive strategies like excessive eating, and inadequate sleep can disrupt hunger-regulating hormones, leading to an increased appetite and cravings for unhealthy foods [10,11]. The consumption of sugar-sweetened beverages and fast foods is identified as an important causative factor for obesity [12,13]. Physical inactivity has been linked to negative health consequences, including an increased risk for obesity, and a negative association between steps/day and adiposity has been documented [14,15]. Screen time has also been linked to obesity through increased eating, decreased sleep, and displacing time a person spends being physically active [16,17].

It has been estimated that one in five children in the US is obese, and the prevalence is highest in Hispanic children [18]. A recent review found that the primary cause of obesity in US children was energy imbalance where calorie intake exceeded calorie expenditure, often combined with a genetic tendency toward weight gain [8]. In China, rapid urbanization has been shown to reshape the lifestyle of ordinary people, and an significant increase in obesity has been documented [19]. The surge was linked not only to low physical exertion, but also to a shift in dietary habits towards more refined grains, highly processed foods, and animal-based products. In a systematic review, it was found that there was an increase in childhood obesity across Brazil, mainly because children were adopting unhealthy routines, often involving poor dietary choices and a lack of PA [20].

Saudi Arabia became the world’s 19th-largest economy after the oil price boom from 2003 to 2013 [21]. Rising prosperity was followed by increased urbanization, which was accompanied by massive changes in dietary and PA habits [22]. Accordingly, these lifestyle-related changes contributed to a surge in obesity prevalence, especially among children. There are 5.34 million children below the age of nine living in Saudi Arabia [23]. They have been born into modernized Saudi culture and have been living a far-from-traditional lifestyle. It was estimated that 34.4% of children in Saudi Arabia are overweight/obese [24]. A recent comparative study by Al-Hussaini et al. found that the rate of obesity in children and adolescents in the capital Riyadh has doubled over the past 10 years [25].

Dental caries is another multifactorial disease that is prevalent amongst children in Saudi Arabia. It was estimated that its national prevalence is 72.1% for the primary dentition, with a mean decayed, missed, filled (dmft) score of 3.93 ± 3.6 [26]. The recent shift from home-cooked meals to empty-calories-rich fast foods, unhealthy snacks, and carbonated beverages could have had an important role in the poor dental health seen in Saudi children, essentially because sugar is often a key ingredient in these so-called “non-food” foods [27,28]. A recent study found that the main source of free sugars in Saudi children was sweetened beverages [29]. Studies that have investigated dietary habits and quality amongst children have found that many children skip breakfast and have a suboptimal intake of micronutrients [30,31]. Furthermore, Saudi children do not consume enough fruit and vegetables, a deficiency that negatively influences their intake of fiber, vitamins, and minerals [32].

Studies have investigated the prevalence of obesity and dental caries in Saudi children, and a positive relationship between the two conditions has been shown in some of the studies [33,34,35,36,37]. Since poor dietary habits are a risk factor for both conditions, the current study was conducted to explore the relationship between diet quality and childhood obesity and dental caries. It also aimed to investigate PA as a risk factor for obesity. To our knowledge, no published studies have investigated the adherence of Saudi children to the Mediterranean diet (MD).

## 2. Materials and Methods

This cross-sectional study is part one of a two-part project. The target population in this part was elementary children. Fifth graders were selected because they are in their mixed dentition stage and are likely capable of completing a questionnaire. There were 48,213 children (50.7% boys and 49.3% girls) enrolled at the fifth-grade level in the city of Jeddah, Saudi Arabia, from which a convenience sample of 300 children (50% boys and 50% girls) was drawn from four private schools (50% boys and 50% girls per school).

The ethics committee at the Faculty of Dentistry at King Abdulaziz University (KAUFD) approved the protocol for this study (064-06-20). Approval to visit schools was obtained from the Saudi Ministry of Education office in Jeddah. Furthermore, brief meetings with the principal of each school took place, in which an explanation of the study was provided and permission to revisit the school to collect data from the children was obtained. Additionally, parental consent forms with a brief description of the study were distributed amongst all fifth-grade children. 

During the next school visit, children who had brought back a signed parental consent form and did not object to participating were enrolled in the study. Inclusion criteria were healthy children between the ages of 8 and 11 years who were in their mixed dentition stage, who were available for examination, and who had the ability and interest to fill out the questionnaire. Data were collected between September 2022 and March 2023.

### 2.1. The Questionnaire

A structured two-section questionnaire was developed based on previous works, as explained below. The forward-backward translation technique was used to reach an accurate Arabic version [38]. The comprehensibility and objectiveness of the survey was examined by five experts at KAUFD (two associate professors in dental public health, an associate professor in pediatric dentistry, a professor in pediatric dentistry, and a professor in medical education). First, face validity was assessed, where each question was rated on a 5-point scale for its importance (not important–very important). Then, content validity was assessed, where each question was rated in four domains—relevance, clarity, simplicity, and ambiguity—on a four-point scale (needs major revision–no need for revision) [39]. The questionnaire in its validated version was pilot-tested on twenty 10-year-olds. The reliability was tested by retesting the twenty children after 2 weeks (Kappa 0.90). These children were not amongst the study participants.

### 2.2. Section I: Diet Evaluation

The Mediterranean Diet Quality Index (KIDMED) was first introduced in 2004 [40]. It is one of the most commonly used scoring systems used to assess adherence to the MD in young populations. Recently an updated version of it was published in accordance with new dietary recommendations [41]. The updated KIDMED questionnaire was used, with the suggested scoring system. Four additional questions were added that were not included in the scoring system. They investigated the children’s consumption of commercial juice, flavored milk, sugar-sweetened carbonated beverages, and potato/corn chips, which are four unhealthy items readily consumed amongst Saudi children.

### 2.3. Section II: Physical Activity

The Physical Activity Questionnaire for Older Children (PAQ-C) was first introduced in 1997 to measure PA in children who are 8–14 years old. It has been used ever since in large-scale surveys. Question 1 of the PAQ-C was modified to be more culturally suitable, where only sports and activities commonly practiced in Saudi Arabia were included (swimming, football, basketball, and running). The same scoring system suggested in the original publication was followed [42]. 

The 5-min questionnaire was self-administered, and most children answered it independently while waiting for the anthropometric measurements to be taken. The questions were read and explained to the children who needed help.

### 2.4. Anthropometric Measurements

Two investigators (general dentists) were trained and calibrated for KAUFD; Kappa score was 0.83 for inter-examiner reliability and 0.86 for intra-examiner reliability.

Height was measured while the children stood barefoot, looking straight forward. The same non-elastic measuring tape was used for all children. Height was rounded to the nearest 0.1 cm. Weight was measured using a digital scale while the kids stood barefoot without jackets and was rounded to the nearest 100 g. Each measurement was taken twice for each child, and the median of these readings was used for the analysis. The BMI for each child was calculated according to the formula weight in kg/height in m^2^. Saudi age-specific and sex-specific BMI percentiles were used to categorize BMI using universal cut-off points [43,44].

### 2.5. Dental Examination

Two calibrated general dentists assessed caries activity using the decayed, missed, and filled scores (dmft/DMFT) in the primary and permanent dentitions, respectively, in accordance with the WHO criteria [45]. Intra-examiner reliability kappa score was 0.90, and inter-examiner reliability kappa score was 0.88. Examination was performed using sterile dental probes and mouth mirrors. Temporary and faulty restorations were marked as decayed. As a courtesy for consenting to enroll their children in the study, a report on caries activity was sent to parents with the contact information of KAUFD’s pediatric dentistry clinics.

### 2.6. Statistical Analyses

Descriptive statistics were used to present the characteristics of the study participants. Means and standard deviations were used to present continuous variables, while frequencies and percentages were used for categorical variables.

The diet score was calculated from the 16-item KIDMED questionnaire. Positive scores were assigned to good dietary behaviors, such as consumption of fruits and vegetables, while negative scores were given to less healthy dietary behaviors, such as sweets and fast food consumption. The total KIDMED score ranged from −4 to 12, with a higher score indicating better dietary behaviors according to the original scoring system. Scores were then categorized into three groups: “Very Poor” (≤3), “Needs Improvement” (4–7), and “Optimal” (≥8). 

Four extra diet questions were added, asking about the daily consumption of chips, bottled juice, flavored milk, and soft drinks. A “Junk Food” score was created based on these variables, which was coded as 0 = no consumption and 1 = consumption. The responses to these questions were combined by summing the values of these questions. Low junk food consumption was considered to be a ≤1 positive answer and high was considered to be ≥2.

A PA score was also calculated by finding the mean of the responses of the different PA questions in the questionnaire in accordance with the original model. We considered means of 1 and 2 to be low activity, 3 to be moderate activity, and 4 and 5 to be high activity.

Normality of dmft/DMFT and BMI percentile were assessed by visually inspecting histograms and by assessing results of skewness and kurtosis tests. Deviation from normality was observed, and therefore, non-parametric tests were used. 

The Kruskal–Wallis test was used to assess associations between KIDMED scores and both health outcomes: dmft/DMFT and BMI percentile. Multiple comparisons were utilized to identify significant differences between groups.

To determine the predictors of BMI percentile, univariate and multivariate linear regression analyses were utilized. The independent variables were age, sex, dmft/DMFT, KIDMED score, junk food score, and PA score. Coefficients and 9% confidence intervals (CI) were presented.

## 3. Results

### General Findings

The study included 300 children with a mean age of 10.6 ± 0.5 years. The majority of the children were Saudi nationals (84.3%). Sex was equally distributed among the participants. The mean weight of the children was 36.4 ± 10.2 Kg, and the height was 139.3 ± 8.1 cm. According to BMI percentile, 2.7% of the children were underweight, 55.3% had a normal weight, and 42% were overweight or obese (Table 1). About 20.67% of children had a dmft/DMFT score of zero.

As illustrated in Table 2, the mean KIDMED score was 3.5 ± 2.9. The median score was 3, with an interquartile range of 1.25 to 5. When the variable was categorized, it was indicated that more than half of the children had a poor diet (58.3%) and 30.7% of the children needed diet improvement, while only 11% achieved an optimal diet.

Table 3 presents the association of KIDMED scores with dmft/DMFT and BMI percentile. Children with very poor KIDMED scores had dmft/DMFT scores (6.2 ± 4.9) which were significantly higher than those who needed improvement (4.9 ± 4.5) and those with optimal diets (3.9 ± 3.4), *p* = 0.012. As for the extra diet questions, low daily chips consumption was associated with lower dmft/DMFT (3.8 ± 3.5), compared with high consumption (5.7 ± 4.8), *p* = 0.049. Lower bottled juice consumption was associated with higher BMI percentile, *p* = 0.027. The BMI percentile did not show significant differences across KIDMED score categories.

Table 4 presents the sports practice and PA characteristics of the participants. Swimming and basketball had the lowest activity levels, with 90.3% and 94% expressing low practice, respectively. Football and running showed slightly higher but also low activity levels. A total of 49% of the children had low activity levels during school breaks, and 28.7% had moderate activity levels. During lunchtime and after school, 58.7% and 58.0% of the participants had low activity levels, respectively. The average PA score is 2.12 ± 0.61.

Table 5 illustrates the predictors of BMI percentile. Older children had lower BMI percentile scores than younger children in the univariate (coefficient = −9.54, 95% CI: −16.83, −2.25) and multivariate (coefficient = −9.35, 95% CI: −17.05, −1.65) analyses. Results of dmft/DMFT illustrate a significantly negative association with BMI percentile in the univariate analyses; however, it became of borderline significance in the multivariate analyses. 

## 4. Discussion

The current cross-sectional study investigated the relationship between childhood obesity and two risk factors, diet quality and PA, as well as the relationship between caries activity and a common risk factor, diet quality. Both obesity and caries were prevalent amongst the 300 elementary school children studied. About 42% of the children were overweight/obese, and 79.33% had current or past caries experience. The mean dmft/DMFT was 5.5 (4.7). Only 11% of children consumed an optimal diet, and only 9% engaged in high PA levels. 

The MD is regarded as a well-balanced diet that is relatively low in saturated fats and is based on the consumption of whole-grain cereals, vegetables and fruit, nuts, seeds, and fish. It includes low to moderate amounts of cheese and yogurt and has a reduced consumption of ultra-processed foods, red meat, and sugars. It has demonstrated a protective role from non-communicable chronic disease [46,47,48]. Although it is not typical for Saudi households, it is considered a healthy diet and has been used in non-Mediterranean studies [48,49,50,51,52,53,54,55]. In the current study, 58.3% of children had very poor diets, and 30.7% needed improvement. Only 11% had optimal diets, which is comparable to the 13.5% shown in studies on Italian children [56]. However, optimal diets were consumed by a larger proposition of children than in the current study in Croatia (20.1%), Spain (46.7% and 31.2%), Greece (43.75%), Chile (30.94%), and Colombia (33%) [49,50,57,58,59,60].

In a recent European systematic review, it was found that poor adherence to the Mediterranean diet (MD) in children (poor diet quality) was associated with higher prevalence of being overweight and obese [61]. Surprisingly, there was no association in the current study between BMI and KIDMED score categories, nor an association between BMI and junk consumption. These results suggest that, within the scope of this research, dietary patterns alone may not have a direct or noticeable impact on BMI. Other factors, such as genetics, PA, or environmental influences, might have a stronger role in determining obesity amongst the studied population, and BMI may not be the most accurate indicator of obesity. These findings highlight the complexity of childhood obesity and the need for a more comprehensive approach when studying its causes. 

On the other hand, children with low adherence to the MD had significantly more dental caries. Furthermore, children with higher daily chips consumption had higher caries activity. Although various genetic, physical, socioeconomic, behavioral, and psychological factors have been associated with the development of dental caries, dietary habits have commonly shown a direct relationship with caries activity [62,63]. A recent 2-year longitudinal study found that children consuming poor diets had significantly more caries experience [64]. Another study found that early childhood caries was linked to a significant dietary imbalance and limited food diversity among Chinese children [65]. That study also revealed that low vegetable intake was associated with a higher risk of caries. Milk and cheese, which are part of the MD, have a preventive role against dental caries [66]. In addition, a high fiber intake was shown to reduce the risk of caries [67]. It can be understood from the current findings that poor adherence to the MD may not only lead to increased consumption of cariogenic foods but could also indicate a deficiency in foods that help prevent dental caries. 

PA is essential for children’s development and the positive impact of regular PA on their health is well documented [68]. Unfortunately, children in the current study barely engaged in PA, and the mean PA score was 2.12 (0.61). Even activities generally common in children, such as running, and sports commonly played by Saudi children, such as football, were minimally practiced, and the majority of children showed low engagement in them (75.33% and 79.00%, respectively). Furthermore, about half the students did not engage in PA during school recess, and less than a third did so moderately. These findings are unusual for children this young, especially amongst boys, and may be related to the hot weather in the afternoon in Jeddah during most of the year or children favoring spending time on their screens [69,70]. Moreover, only 13.33% engaged in high activity after school, and about 60% were barely active. On weekends, 60.33% of children were barely active and only a minority were engaged in high activity. It seems from these findings that the culture of sports is not strongly embedded in the studied population, and children spent their recess, after-school time, and weekends being inactive. This is in accordance with unfortunate findings from a recent review that found a consistently inadequate level of PA among children on a global level [71].

International and local studies have shown that children who engage in sports or are active have lower BMI percentiles and body fat [72,73,74,75,76]. In the current study, however, there was no association between the amount of PA and the different BMI categories. A plausible explanation is that most children did not participate in PA; thus, it was difficult to detect associations with such a low PA prevalence. Another explanation is that BMI was used as an indicator for obesity in the current study; results may have differed had other measures like body fat percentage been measured. 

Childhood is a stage best recognized for its malleability. The elementary school years are amongst the most impressionable years of a child’s life [77]. Dietary habits are formed in childhood and are introductory to habits in adolescence and adulthood [78]. Furthermore, children are active by nature, and research has shown that this activity declines as they grow [71]. For this, it is crucial to instill as many positive behaviors as possible early in life which could have a long-lasting positive impact on the child’s health. Children in the current study did not consume optimal diets and were not physically active. Although the findings may not be generalizable to Saudi children, they mandate prompt and effective interventions to limit negative consequences on health while change is easier and less costly. School-based educational programs are encouraged for mass delivery. Schools are encouraged to provide healthy food options in their canteens and identify ways to encourage PA during recess. Furthermore, the promotion and facilitation of healthy lifestyles are encouraged on a public health level. The findings also encourage the establishment of a healthy Saudi diet reference which could be useful as a guideline for the public and for future research.

The study is not without limitations. Although the questionnaire in two parts has been previously used with children, current participants may have encountered difficulty recalling information accurately and may have put responses perceived as more desirable or socially acceptable which may have affected the accuracy of their responses. An updated version of KIDMED was published by López-Gajardo et al. in 2022, but data collection had already been in progress [79]. Moreover, the study used adherence to the MD as a benchmark for consuming a healthy diet, while it is not commonly consumed in Saudi households. However, it was used due to the lack of a healthy Saudi diet reference alternative. On the other hand, there were notable strengths to the study. First, the questionnaire that was used was thoroughly validated by experts in the field. Furthermore, the questionnaire was culturally adapted, and modifications were made to better collect relevant data without compromising it or its scoring system. The methodology is thorough and easily reproducible. Last, the study is the first to shed light on the adherence of Saudi children to one of the healthiest diets, the MD, which could be a niche for future studies.

## 5. Conclusions

In conclusion, children consumed poor diets, and there was a positive association with dental caries. However, no association was found between diet quality and obesity, nor between PA level and obesity. 

Future research should explore these relationships in more detail by using larger and more diverse sample populations and incorporating more precise measurements of dietary intake, physical activity, and other lifestyle factors. Additionally, considering the interaction of genetic predisposition and environmental factors may help uncover subtler associations and potentially uncover relationships not evident in the current study. 

## Figures and Tables

**Table 1:** Characteristics of the children (n = 300).

Characteristic	N (%)
**Age**, mean (SD)	10.6 (0.5)
**Nationality**	
Saudi	253 (84.3)
Non-Saudi	47 (15.7)
**Sex**	
Male	150 (50)
Female	150 (50)
**Weight**, mean (SD)	36.4 (10.2)
**Height**, mean (SD)	139.3 (8.1)
**BMI**, mean (SD)	18.6 (4.3)
**BMI percentile**, mean (SD)	61.8 (32.5)
**BMI percentile**, categorical	
Underweight	8 (2.7)
Normal	166 (55.3)
Overweight/obese	126 (42.0)
**Decayed**, mean (SD)	4.3 (4.4)
**dmft/DMFT**, mean (SD)	5.5 (4.7)

**Table 2:** Descriptives of KIDMED score among the participants.

KIDMED Score Statistics	Value
Mean (SD)	3.5 (2.9)
Median (IQR)	3 (1.25, 5)
Range	−2, 12
Categories	N (%)
Very Poor (≤ 3)	175 (58.3)
Needs Improvement (4–7)	92 (30.7)
Optimal (≥8)	33 (11.0)

**Table 3:** Distribution of KIDMED scores and associations with DMFT and BMI percentile.

Item	d/D. Mean (SD)	*p*-Value	Dmft/DMFT, Mean (SD)	*p*-Value	Multiple Comparisons	BMI Percentile	*p*-Value
**KIDMED score**							
Very poor	4.6 (4.6)	0.227 ^	6.2 (4.9)	0.012 ^	V vs. N * V vs. O * N vs. O	62.6 (32.1)	0.791 ^
Needs improvement	4.1 (4.4)		4.9 (4.5)			61.9 (33.6)	
Optimal	2.8 (2.6)		3.9 (3.4)			57.3 (32.5)	
**Daily chips consumption**							
No	2.7 (3.1)	0.066 ∅	3.8 (3.5)	0.049 ∅	---	61.3 (33.8)	0.996 ∅
Yes	4.4 (4.5)		5.7 (4.8)			61.8 (32.4)	
**Daily bottled juice consumption**							
No	4.2 (4.6)	0.393 ∅	5.9 (4.9)	0.349 ∅	---	65.7 (31.6)	0.027 ∅
Yes	4.3 (4.1)		5.2 (4.4)			57.9 (33.0)	
**Daily flavored milk consumption**							
No	4.1 (4.4)	0.599 ∅	5.4 (4.8)	0.563 ∅	---	65.7 (31.6)	0.088 ∅
Yes	4.3 (4.3)		5.6 (4.6)			59. (33.3)	
**Daily soft drink consumption**							
No	2.9 (2.9)	0.481 ∅	3.5 (3.4)	0.185 ∅	---	52.2 (32.7)	0.404 ∅
Yes	4.3 (4.4)		5.6 (4.7)			62.2 (32.5)	

V = very poor, N = needs improvement, O = optimal. * Statistically significant multiple comparisons at 0.05 level. ^ Kruskal–Wallis test, ∅ Wilcoxon rank sum test.

**Table 4:** Distribution of sports and PA among the participants.

Variable	N (%)
**Have you done any of the following activities in the past 7 days (last week)? If yes, how many times?**	
**Swimming**	
Low activity	271 (90.33)
Moderate activity	9 (3.00)
High activity	20 (6.67)
**Football**	
Low activity	237 (79.00)
Moderate activity	53 (17.67)
High activity	10 (3.33)
**Basketball**	
Low activity	282 (94.00)
Moderate activity	13 (4.33)
High activity	5 (1.67)
**Running**	
Low activity	226 (75.33)
Moderate activity	34 (11.33)
High activity	40 (13.33)
**In the last 7 days, what did you do most of the time at school recess?**	
Low activity	146 (48.67)
Moderate activity	86 (28.67)
High activity	68 (22.67)
**In the last 7 days, what did you normally do at lunch (besides eating lunch)?**	
Low activity	176 (58.67)
Moderate activity	71 (23.67)
High activity	53 (17.67)
**In the last 7 days, on how many days did you play sports, dance, or play games in which you were very active after school?**	
Low activity	174 (58.00)
Moderate activity	86 (28.67)
High activity	40 (13.33)
**During the last weekend, how many times did you play sports, dance, or play games in which you were very active?**	
Low activity	181 (60.33)
Moderate activity	86 (28.67)
High activity	33 (11.00)
**How do you describe yourself in terms of PA last week?**	
Low activity	94 (31.33)
Moderate activity	173 (57.67)
High activity	33 (11.00)
**PA Score**	
Mean (SD)	2.12 (0.61)
Low PA (mean of 1–2)	47%
Medium PA (mean of 3)	44%
High PA (mean of 4–5)	9%

**Table 5:** Predictors of BMI percentile.

Variable	Univariate Coefficient (95% CI)	Multivariate Coefficient (95% CI)
**Age**, mean (SD)	−9.54 (−16.83, −2.25)	−9.35 (−17.05, −1.65)
**Sex**		
Male	1.0 (Reference)	1.0 (Reference)
Female	1.01 (−6.40, 8.41)	1.95 (−8.78, 12.68)
**dmft/DMFT**	−0.91 (−1.69, −0.12)	−0.83 (−1.67, 0.01)
**KIDMED score**		
Optimal	1.0 (Reference)	1.0 (Reference)
Needs improvement	4.60 (−8.42, 17.61)	5.33 (−7.72, 18.38)
Very poor	5.27 (−6.90, 17.45)	11.00 (−3.41, 25.42)
**Junk food score**		
Low junk food consumption (0–1)	1.0 (Reference)	1.0 (Reference)
High junk food consumption (2–4)	6.94 (−1.46, 15.34)	4.93 (−3.46, 13.32)
**PA score**	4.77 (−1.33, 10.9)	6.42 (−0.55, 13.40)

## Data Availability

All data generated for this study are available from the corresponding author upon reasonable request. The data are not publicly available to protect the data privacy and restrict unauthorized use.

## Abbreviations

**BMI:** Body Mass Index, **CI:** Confidence Interval, **dmft/DMFT:** decayed, missed filled score in the primary and permanent teeth, **DSR**: Deanship of Scientific Research, **KAUFD:** Faculty of Dentistry at King Abdulaziz University, **KIDMED:** The Mediterranean Diet Quality Index, **MD:** Mediterranean Diet, **PA:** Physical Activity, **PAQ-C:** Physical Activity Questionnaire for Older Children, **SD**: Standard Deviation; **WHO:** World Health Organization.
